# Supplementary figures and images for: In vivo coupling of dendritic complexity with presynaptic density in primary tauopathies
Source: Neurobiol Aging. 2021 May;101:187–98. doi: 10.1016/j.neurobiolaging.2021.01.016 (PMC8209289; doi:10.1016/j.neurobiolaging.2021.01.016)

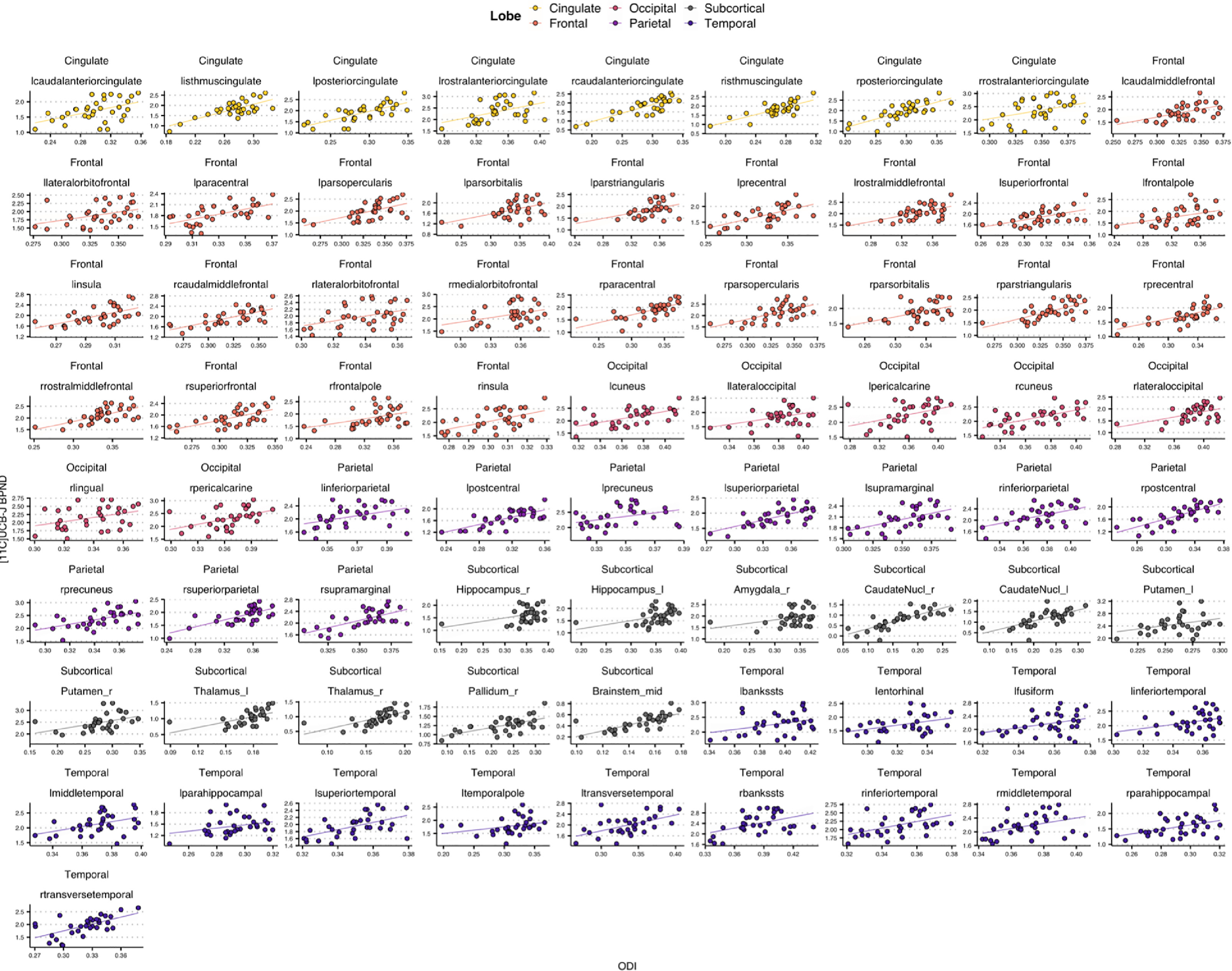

Supplement: Supplementary file 1 [file mmc1.jpg]
